# From scientific arguments to scepticism: Humans’ place in the Greenhouse

**DOI:** 10.1177/09636625211035624

**Published:** 2021-09-02

**Authors:** Anaïs Augé

**Affiliations:** University of East Anglia, UK

**Keywords:** greenhouse effect, media, online forum, scepticism, science

## Abstract

This article investigates the different roles attributed to humanity in the climate change debate, through the depiction of the *greenhouse effect*. Our hypothesis is that the stance associated with different genres will not only demonstrate different conceptualisations of the greenhouse effect but also convey different views on humans’ capacity (or lack of capacity) to mitigate climate change. The corpus under study is composed of texts pertaining to three genres which display particular viewpoints: scientific papers present a documented view on the phenomenon, online forum discussions present exchanges between users who endorse or question particular characteristics of the *Greenhouse*, and sceptical newspaper articles explicitly deny the existence of an anthropogenic phenomenon. Through a corpus-based, cognitive and pragmatic analysis of the metaphorical expression *greenhouse effect*, the research shows that humans’ place(s) in the *Greenhouse* is a significant part of environmental argumentative strategies.

## 1. Introduction

This article proposes an investigation of the varying use of the metaphorical expression *greenhouse effect* in climate change discourse. This particular metaphor is in focus because the expression *greenhouse effect* may describe either a concept that is related to environmental disruption (i.e. anthropogenic climate change) or a concept that is related to a natural process enabling life on Earth. Therefore, I distinguish the metaphor *greenhouse effect* from other climate change metaphors such as *carbon footprint* (Koteyko, 2010), *runaway effect* (Van Der Hel et al., 2018) or *war on pollution* (Atanasova and Koteyko, 2017). Indeed, these metaphors are all associated with the phenomenon of climate change and human alteration of the environment. In this article, I thus ask how metaphor users rely on the varying meaning of the expression *greenhouse effect* depending on the stance adopted in the texts. My hypothesis is that, while scientists may draw a distinction between a natural and an anthropogenic greenhouse effect, sceptical arguments may merge these two concepts to dispute the idea of an anthropogenic phenomenon (more details below).

The arguments analysed below are related to the role of humanity in the modification of the *Greenhouse*. In the corpus under study, we see that sceptical strategies can either deny humans’ responsibility or these may ignore the association between the *greenhouse effect* and climate change. These arguments attribute different roles to humans: humans can be depicted as the BUILDERS of a dangerous *Greenhouse* or as the DESTROYERS of the natural *Greenhouse*, or they can only be described as the CONTENT of the *Greenhouse*, deprived of any agency.

To appreciate the association between different stances and different perspectives on humanity in the *Greenhouse*, this article focuses on the arguments reported in three genres. These three genres present a graduation from objectivity, argumentation and scepticism. First, scientific papers represent documented views on the *greenhouse effect* and objective viewpoint is an essential criterion for scientific legitimacy (Knudsen, 2015). Second, online forum discussions highlight the characteristics of the *greenhouse effect*, which can be questioned or disputed. Third, sceptical media offer an explicit bias on the *greenhouse effect* which aims at convincing readers that the anthropogenic *greenhouse effect* is not real.

Relying on cognitive linguistics, pragmatics and discourse analysis, I demonstrate how a scientific metaphor can convey opposite arguments in different contexts. The following section will explain the significance of metaphors in discourses describing a scientific topic, I then provide more information about my corpus and my methodology, and I present the different uses of the metaphor in the three genres. The discussion will highlight the interrelation between the different roles attributed to humans and different stances.

## 2. Climate change and metaphors

### What is a metaphor

My review of existing literature about climate change metaphors must first establish the significance of metaphors as a figurative device in discourse. From a cognitive perspective, a metaphor involves two conceptual domains: the target domain represents the literal concept which may be too complex or too abstract to be described literally. This target domain is associated with a source domain that represents a figurative, ‘alien’ concept which is more concrete and which is more familiar to the metaphor recipient (Lakoff, 1993 [1979]). For example, the metaphorical compound *carbon footprint* depicts the impact and extent of carbon pollution (i.e. the target domain) with reference to a concrete, visible *footprint* (i.e. the source domain). The presence of this compound in a plurality of texts about climate change (Koteyko, 2010) makes it easier for metaphor recipients to understand and quantify the impact of carbon pollution.

Pragmatic views on metaphors have established that the association between the two domains (called a ‘mapping’; Lakoff, 1993 [1979]) is performed through a transfer of characteristics from one domain to another (Glucksberg, 2001). While the cognitive view does not necessarily tackle the significance of metaphors in context, pragmatics and discourse analysis have demonstrated that the meaning of the metaphor varies depending on the context in which it is used. Therefore, the target domain of the metaphor will be perceived according to certain characteristics shared with the source domain which are relevant in a particular context. For example, depending on the topic discussed in the texts, the *carbon footprint* aforementioned may refer to a *large footprint* or to a *small footprint* (Augé, 2022).

The metaphorical meaning can be exploited and disputed to promote various arguments. These various arguments reveal the presence of metaphor scenarios in discourse (Musolff, 2016). Scenarios include metaphorical expressions in argumentative discourses. They highlight the way the source domain (i.e. the concrete concept) can be perceived from different viewpoints to influence recipients’ opinions on a topic (Musolff, 2016: 30–31). For example, while the metaphorical compound *carbon footprint* is a significant concept in climate change discourse, this compound can be adapted to criticise specific polluters, attributing the *footprint* to a *Carbon Bigfoot* (Koteyko et al., 2009: 43). The expression drives the recipients’ attention towards polluters instead of pollution and it conveys particular arguments regarding the responsibility of polluters.

In the next section, I offer an overview of the existing findings related to climate change metaphors.

### Climate change discourse

Through the mapping of a complex target domain and a concrete source domain, metaphors play an essential role in discourses about science (Knudsen, 2015). In the context of climate change, these help to describe environmental concepts to a wide readership with limited scientific background (as opposed to a scientific readership). Since climate change is a global dangerous phenomenon, populations’ understanding of scientific concepts is essential to convince them to reduce pollution. Metaphors can help in this process through the reliance on familiar words to explain concepts such as the amount and impact of pollution, extreme weather events and their causes, or the evolution of climate. It is thus unsurprising to observe a wide variety of metaphors in climate change discourse: the *tipping point* metaphor describes the evolution of climate change towards a point where the phenomenon is out of control (Van der Hel et al., 2018), the *carbon footprint* describes the amount and impact of carbon pollution (Koteyko, 2010; Nerlich and Hellsten, 2014), *green-washing* describes economically biased processes that present marketed products as environmentally friendly (Pérez-Sobrino, 2013), and the personification *Mother Earth* describes humans’ dependence on nature (Augé, 2019).

Since climate change is still a disputed topic associated with a plurality of discourses (scientific, political, journalistic or environmentalist) and a plurality of opinions, related metaphors are accordingly involved in the promotion of various arguments. For instance, scholars demonstrate how WAR metaphors associated with climate change can convince recipients of the urgency to find solutions, as in: ‘How to *win the war* on global warming?’ (Mangat and Dalby, 2018: 3). Atanasova and Koteyko (2017) show that this metaphor is predominantly associated with environmentalist arguments in newspapers. In addition, Flusberg et al.’s (2017) survey shows that WAR metaphors are more likely to convince recipients to act upon climate change as opposed to other metaphors such as RACE metaphors, as in ‘When will Americans *go after* excessive energy use and *surge ahead* on problems?’ (p. 772). Indeed, the metaphorical idea of *winning a race* comprises positive, yet non-essential characteristics compared with the metaphorical idea of *winning a war*, which comprises necessary characteristics for survival (Flusberg et al., 2017: 772). Alternatively, RELIGION metaphors in climate change discourse have been shown to promote scepticism in the media, as in the following: ‘They (G8 leaders) are like *medieval preachers* proclaiming to baying crowds that the end of the world in nigh’ (Atanasova and Koteyko, 2017: 460). Nerlich (2010) establishes that RELIGION metaphors represent a significant part of climate change discourse when these focus on scandals like the scandal of the Climategate.^1^ The complexity of the problem and the existing scientific uncertainties regarding climatic evolution were mapped with the uncertainty regarding religious dogma. However, other scholars demonstrate that RELIGION metaphors have been used to dispute such sceptical arguments to present scientists as *prophets* capable of interpreting signs (Foust and Murphy, 2009: 153–156). Nay and Brunson’s (2013) survey demonstrates that MEDICINE metaphors may also effectively convince recipients to protect the forests (p. 165), as in the following: ‘An ecological system is *healthy* and free from *distress syndrome* if it is stable and sustainable’ (Ross et al., 1997: 119). MEDICINE metaphors can promote compassion among recipients who can interpret environmental disruption according to their own experience of sickness (Augé, 2021).

While I acknowledge the significant contribution of existing research about climate change metaphors, this overview suggests that metaphorical environmentalist arguments have mainly been observed in the use of non-specific metaphors such as WAR, RELIGION, RACE and MEDICINE. The next section will focus on the particularities of the *greenhouse effect* metaphor.

### The greenhouse effect

With attention paid to the *greenhouse effect*, existing literature demonstrates that this concept is constitutive of climate change scientific theory (Nerlich and Hellsten, 2014). The source domain GREENHOUSE comprises a wide variety of characteristics, which can map with climate change: the concept *Greenhouse* describes a container in which specific temperatures are to be kept to enable particular plants to grow. Romaine (1996) relates the expression to the mapping EARTH AS A CONTAINER (p. 181). The particularities of the source domain GREENHOUSE involve an additional mapping emerging from the EARTH-CONTAINER, which identifies HUMANS AS PLANTS (i.e. the plants are the typical content of the Greenhouse; Romaine, 1996: 181). Nerlich and Hellsten (2014) demonstrate how the source domain GREENHOUSE helps metaphor recipients to see a concrete representation of the impact of pollution (p. 28). Deignan et al. (2019) indicate that the *greenhouse effect* metaphor is used by different discourse communities. They show that this metaphor seldom occurs in academic articles about climate change while educational texts and interviewed students use this particular metaphor more frequently (Deignan et al., 2019: 385–388). They also note that the *greenhouse effect* metaphor may lead to misunderstandings. The interviewed students referred to the *greenhouse effect* as a thin layer around the planet which does not let heat out, while *greenhouse gases* are rather ‘dispersed’ in the atmosphere, according to the linguists’ academic corpus (Deignan et al., 2019: 394). Such a misunderstanding of the scientific concept is supplemented by a ‘domestication’ process observed in the media, where climate change is misrepresented as a local phenomenon (Brown, et al. 2011: 664–665; Olausson, 2009).

This article offers a different viewpoint on the *greenhouse effect*. I see that existing literature has mostly focused on its explanatory function. Here, I investigate the argumentative role of this metaphor in scientific discussions, online forum discussions and sceptical media commentaries. I aim at showing that the *greenhouse effect* metaphor is not only a ‘technical term’ but has a significant role to play in the promotion of arguments about anthropogenic climate change.

## 3. Methodology

To investigate the different arguments related to the metaphorical expression *greenhouse effect*, I build a corpus of texts composed of scientific articles, online discussions and newspapers.

To access scientific articles, I relied on the *Web of Science* (n.d.), which gives access to a plurality of research articles. I limited my selection to papers published in the journal *Nature* released between 1990 and 2019. *Nature* has been selected for this study to discuss exemplary uses of the metaphor in a scientific context. This journal has been of particular interest, as opposed to other scientific journals like *Science* or *Climate Change*, because I noticed many references to these papers in the media (see below) and because my research on *Web of Science* has demonstrated that *Nature* is the journal that most frequently relies on the expressions ‘climate change’ and ‘global warming’ in the titles of the associated papers. Such explicit references to the phenomenon are helpful to non-scientists to rapidly identify the main topic discussed in the articles which can, then, be included in the corpus. To access these articles, I used particular keywords: ‘climate change’ OR ‘global warming’ as the topic, and ‘greenhouse effect’ as a term included in these articles. This procedure resulted in a corpus composed of 153 scientific articles.

Online discussions about the environment were selected from the electronic corpus provider *SketchEngine* (Kilgarriff, 2014). *SketchEngine* is an online corpus provider, which gives access to different electronic corpora of texts produced in different languages and different contexts (i.e. general contexts, political contexts, scientific contexts, historical contexts or online discussions). These corpora allow the analyst to search for a particular search term (here, ‘greenhouse effect’) to observe a plurality of data which rely on this term in different contexts. Among the available electronic corpora, I selected the corpus *EnTenTen* which is an electronic corpus of data produced in English. The data comprised in the corpus *EnTenTen* have all been collected from the Internet (e.g. blogs, forums, online commentaries, advertisements). *EnTenTen* gathers data, which have been produced from 2008 to 2018 (latest version of the corpus produced in 2015). Thanks to the functions provided by *SketchEngine*, the researcher may focus on data collected during a specific time, in a specific language and on a specific online source. For the purpose of my research, I discarded the data occurring in specialised contexts such as scientific blogs as well as data occurring in online press releases (explicitly identified as such in *SketchEngine* advanced search tools). I thus focused on the data produced by ‘lay’ people, that is, general blogs, online commentaries and forums (as indicated in the sources provided by *SketchEngine*). I first performed a search using the function *WordSketch*, which presents the most frequent collocates of the search term (here, ‘greenhouse effect’; see Table 1). This research yielded 8433 occurrences in the electronic corpus, which have been automatically classified according to the collocates. These collocations help to see automatically generated categories of the different uses of the search term. These categories thus offer a first overview of the different contexts and, more precisely, the different concepts with which ‘the greenhouse effect’ can be associated. I then performed a more intensive search, paying attention to the contextual information provided for each type of collocations. This contextual information helped me to establish the source from which the data have been extracted (blogs, commentaries, forums, identified in the ‘information’ settings available for each data), with attention paid to the characteristics of the source (I can infer that scientists or journalists may also rely on blogs, commentaries and forums) to avoid including data that would be similar to the data I investigate in the other genres (scientific papers and media). In addition, this information warrants the assumption that the expression is used in a context associated with climate change, with particular attention paid to specific words indicating a contextual link, that is, ‘pollution’, ‘environment’, ‘climate’, ‘weather’ or ‘global’. When doubts persisted regarding the context of use, the decision was not to include the related data in my corpus. An overview of the collocations provided by *EnTenTen* is displayed in Table 1.

**Table 1.** Overview of the collocates of ‘greenhouse effect’ (*EnTenTen*).

Modifiers	Modified by ‘greenhouse effect’	Object of	Subject of	‘greenhouse effect’ and/or	Qualified by	‘greenhouse effect’ is a
runaway	depletion	counteract	trap	warming	real	phenomenon
radiative	acidification	mitigate	warm	albedo	wrong	science
enhanced	aerosol	exacerbate	cause	depletion	strong	process
anthropogenic	warming	amplify	exist	smog		cause
atmospheric	Earth	intensify		ozone		time
CO_2_	dioxide	worsen		acidification		problem
warming	rain	aggravate		CO_2_		thing
Gas	pollution	combat		aerosol		
manmade		counter		dioxide		
Human-induced		cause		pollution		
		reverse		rain		
		accelerate		radiation		

Newspaper articles have been retrieved from the database *Nexis* (n.d.). This database allows researchers to access articles from different countries and produced over a 40-year timespan. For the present purpose, I selected English language newspapers with the keywords ‘climate change’ OR ‘global warming’ AND ‘greenhouse effect’. The period of publication starts in 1990 and ends in 2019. I focused on English language newspapers because I do not aim at establishing a link between environmental arguments and cultures. I thus selected newspapers displaying texts produced in the English language, although admittedly United Kingdom, United States or Australian stances may highlight cultural variations. In addition, I selected articles whose headlines explicitly showed a sceptical stance towards climate change. For example, I selected articles which described the phenomenon or associated decisions as a ‘scam’, a ‘con-trick’, ‘hot air’, a ‘fake’ news, or a ‘wrong’ claim. Instances of such sceptical arguments can be best described in headlines such as ‘The climate change scam: what should I believe and why’ (*News Wire*, US, 24 September 2015). This selection resulted in the inclusion of 297 articles in my corpus.

To interpret the metaphorical expression in context, I used the Metaphor Identification Procedure VU University Amsterdam (MIPVU) (Steen et al., 2010). MIPVU proposes four steps to determine whether an expression can be established as metaphorical:

1. Reading the text

2. Selecting lexical units

3. Interpreting these units in context and searching for a more ‘basic’ meaning in other contexts

4. Interpreting the ‘basic’ meaning in the context under study. (Steen et al., 2010: 4–6)

The emphasis on the role played by the communication level on the interpretation of a metaphorical expression required additional steps: the researcher can consult dictionary entries to determine the referential meaning of the utterance in context (Reijnierse et al., 2018).

Once I distinguished metaphorical occurrences of *greenhouse effect* from non-metaphorical occurrences in our corpus, I observed the co-text of the occurrences. This initial observation permitted an overview of the role played by humans within the mapping EARTH AS A GREENHOUSE in scientific papers, online discussions and sceptical newspaper articles.

Even though the timespan of the publication of texts and the number of texts and occurrences significantly vary from one genre to another, I emphasise that the scope of this research is corpus-based (Tognini-Bonelli, 2001): I aim at presenting various uses of the metaphor *greenhouse effect* in the different genres to observe the roles attributed to humans, but our findings do not tackle the overall use of the metaphor in the totality of my corpus. Therefore, when an occurrence was established as metaphorical but did not promote particular argument or was not explicitly related to humans’ contribution, I did not include it in my corpus. In addition, when doubt persisted regarding the interpretation, the occurrence was not included either. Following this selection, I obtained a corpus of 71 occurrences in scientific papers, 392 occurrences in online discussions and 95 metaphorical occurrences in newspapers. The findings presented in the following sections highlight how the place of humans within the *Greenhouse* may differ depending on the arguments promoted in each text.

## 4. Scientific arguments

The papers selected from the journal *Nature* show a distinction between the natural *greenhouse effect* and its anthropogenic version related to climate change. We see that ‘the greenhouse effect’ can be explicitly characterised with particular qualifications: these rely on adjectives such as ‘natural’, ‘anthropogenic’, ‘human-made’, ‘beneficial’ or ‘threatening’. These adjectives highlight that the concept *greenhouse effect* is a unique effect that is progressively transformed by human activities. Such a transformation is adequately described in the following example:

(1) Although models (and simple theory) indicate that such a change, which would increase mid-latitude land precipitation, might be expected to accompany *an anthropogenic greenhouse warming*, most models seem to underestimate the magnitude of this circulation change. (. . .) Physically, therefore, it has long seemed plausible that the distribution of relative humidity would remain roughly constant under climate change, in which case the Clausius–Clapeyron relation implies that specific humidity would increase roughly exponentially with temperature. (*Nature*, volume 419, 224–232 (12 September 2002), Myles R. Allen and William J. Ingram)

In extract (1), the scientists refer to a ‘greenhouse warming’ instead of a ‘greenhouse effect’. While both expressions depict a global system that warms the planet, the former phrase emphasises the negative impact of the warming. Indeed, while the *greenhouse effect* is an expression which can be applied to both natural and unnatural processes, *greenhouse warming* expresses the increase in temperatures, which differs from the temperatures provided by *greenhouse effect*. The addition of the adjective ‘anthropogenic’ identifies the human origin of this increase. The scientists thereby linguistically emphasise the (negative) evolution of the *greenhouse effect* which is referred to by two distinct expressions to differentiate the warming processes. Hence, humans’ active role is perceived as the origin of the ‘change’, and this change comes with negative characteristics explained in the remainder of the extract: ‘increase mid-latitude land precipitation’, ‘specific humidity would increase roughly exponentially with temperature’. Scientists attest that human activities have not only increased warming but also modified the functioning of the *greenhouse effect* to the point that this effect is now characterised as an ‘anthropogenic warming’.

In other extracts, the scientists focus on the identification of gases and pollution as the MATERIAL of the human-made *Greenhouse*. In such cases, humans’ role is even more significant as humans become the BUILDERS of the unnatural *Greenhouse* or the DESTROYERS of the natural *Greenhouse*. These characterisations can be observed in the extract presented below:

(2) Previous studies have concluded that climate change owing to *increasing greenhouse-gas concentrations* will have *a detrimental effect on the ozone layer* over the next few decades (. . .) We emphasize, however, that while CFC levels remain artificially high, the *ozone layer will continue to be vulnerable to the enhanced destruction resulting from a greenhouse cooling of the stratosphere*. (*Nature*, volume 410, 799–802 (12 April 2001), Neal Butchart and Adam A. Scaife)

In extract (2), the scientists describe ‘greenhouse-gas concentrations’ as the origin of climate change. Although human activities are not explicitly mentioned, the link established between the concentrations and climate change shows that the expression refers to the unnatural *Greenhouse*. In such cases, humans are not only seen as the BUILDERS OF THE UNNATURAL GREENHOUSE but also as the DESTROYERS OF THE NATURAL GREENHOUSE. Indeed, while humans’ addition of gases is modifying the GREENHOUSE-EARTH (‘owing to increasing greenhouse-gas concentrations’; ‘CFC levels remain artificially high’), this addition is also described as destroying some components of the NATURAL GREENHOUSE, the ozone layer. The GREENHOUSE is here mapped to another target concept: the cooling stratosphere, which indicates that the UNNATURAL GREENHOUSE also causes the cooling of components. Hence, the addition of MATERIAL (CFCs) transforms not only the characteristics of the GREENHOUSE-ATMOSPHERE but also the characteristics of the GREENHOUSE-STRATOSPHERE. Therefore, humans’ active role in the GREENHOUSE is emphasised as they implicitly perform the dual functions of BUILDERS and DESTROYERS of the GREENHOUSES.

In different articles, scientists rely on the metaphor to describe the evolution of the *greenhouse effect*. Since the exact characteristics of this evolution are linked with significant uncertainties, scientists dispute existing claims regarding the need to prepare for an apocalyptic *Greenhouse* world (Foust and Murphy, 2009) without adopting preventive solutions. The metaphor EARTH AS A HEATED CONTAINER aims at illustrating an imaginary state of future climate, as in:

(3) But I am not aware of any scientist or environmental activist who have ever used the phrase *‘runaway global warming’* in the context of Earth (the jargon is usually reserved for *the oven-like super-greenhouse effect on Venus*); and, needless to say these *dissembling activists and scientist-hysterics* go unnamed and uncited in Parsons’ text. (*Nature*, volume 381, 384–386 (30 May 1996), Stephen H. Schneider)^2^

In extract (3), the scientist describes the *greenhouse effect* as an OVEN to depict the warmth on the planet Venus, which prevents life on this planet. This conceptualisation aims at criticising Edward Parson, a Professor of Law and Environment.^3^ This criticism is a reply to Parson’s stance about (supposed) scientific characterisation of the *greenhouse effect* on Earth as a RUNAWAY effect.

This scientific characterisation is denied by Stephen Schneider who attributes a RUNAWAY characteristic to the climatic conditions of Venus. He emphasises the impossibility of applying this characteristic to the Earth through the use of the source domain OVEN. This source domain is effective in illustrating a very high degree of heat, which is fatal to humans. Since the warming on Earth does not prevent human life, the temperatures cannot be equated to OVEN-LIKE temperatures and the *greenhouse effect* cannot be described as a RUNAWAY effect.

The scientist relies on exaggeration to contradict Parson’s claims. He also questions the existence of ‘dissembling activists and scientists-hysterics’ who supposedly coined this analogical qualification. The characterisation of these scientists as ‘hysterics’ emphasises the absurdity of the comparison between the climatic conditions on Venus and the conditions on Earth.

These three examples from *Nature* show that scientists give a prevalent role to humanity in the *Greenhouse*. This role is associated with increasing warmth within the CONTAINER and We see a criticism related to human activities: humans are described as BUILDING a *Greenhouse* that endangers their life while they also DESTROY a *Greenhouse* that offer(ed) them a pleasant environment to live in. We also see that scientific stance may dispute humans’ lack of capacity to act upon the *greenhouse effect*: characterisation of humans as the passive victims ‘cooked in an oven’ is explicitly criticised to promote existing solutions. In the following section, I will study the uses of the metaphor in online discussions.

## 5. Arguments from online discussions

The occurrences of the metaphorical expression *greenhouse effect* in online discussions offer an overview of the characteristics of the concept, which can be debated by online users. We see that debates about the g*reenhouse effect* can encompass humans’ responsibility to mitigate the effects. We also see how sceptical stances can be moderated or contradicted through online exchanges. Although I cannot always access the full exchanges from which the selected data originate, the varying use of the metaphor can highlight the negotiation of the characteristics of the *Greenhouse*. For instance, the example below shows that sceptical stance can be associated with a promotion of humans’ responsibility:

(4) I love the minimalistic idea of living with less. I believe that the best way to save on home expenses is to live in a house in the country. You can produce your own organic food, your own fuel for heating, your own power from alternative sources like solar panels. While *I don’t believe in such bullcrap like human-induced greenhouse effect*, I want to reduce my impact on the environment. I want to use as little fossil fuels as possible, having my own plot of land will allow me to produce my own biomass. (*EnTenTen*, token: 13073468485)

In extract (4), the metaphor user denies the ‘human-induced’ characteristics of the *greenhouse effect*. Here, the alteration of temperatures is not perceived as a clue of the transformation of the *Greenhouse*. Indeed, climatic changes are not mentioned to give prevalence to the role of humans within the *Greenhouse*. While the stance of the extract is sceptical ( ‘bullcrap’), the metaphor user presents environmental actions as a reasonable human behaviour. Cutting down fossil fuels and living in a sustainable manner are actions which can be assimilated, in the extract, to CLEANING-SANITISING ONE’S HABITAT. I can observe an argumentative strategy which places humans in the role of the inhabitants of the shared *Greenhouse*. This conceptualisation implies that while the anthropogenic cause of climate change is denied, humans are yet attributed the responsibility to take care of the planet. Such a responsibility can be emphasised further in the discussions, especially when the metaphor is used in a politically oriented context showing the users’ opinions on environmental decisions. This emphasis is observed in the example below:

(5) In addition if *carbon dioxide contributes 20% to the terrestrial greenhouse effect*, then what *a waste of time a carbon tax will be* in Australia, especially if the ‘others’ don’t follow suit. It is fiction to believe we will set an example and the others will follow, Australia already tried that with free trade and level playing fields; as a result our manufacturing industries are a basket case and agriculture is following close behind. (*EnTenTen*, token: 2675720970)

In extract (5), the metaphor user refers to the conceptualisation of humans as the BUILDERS of the unnatural *Greenhouse* through a criticism related to the ‘carbon tax’. This tax aims at forcing carbon emitters to financially contribute to climate change mitigation. However, the metaphor user is critical of such a contribution: he or she highlights that the identification of HUMANS AS BUILDERS only holds because of humans’ carbon emissions. Yet, he or she emphasises that carbon represents a minor element of the MATERIAL of the *Greenhouse* (‘20%’). Hence, in view of this percentage, the association between HUMANS-BUILDERS and carbon emissions implies that humans cannot be identified as the main BUILDERS. An additional argument refers to humanity as the global CONTENT OF THE GREENHOUSE to promote global responsibility. This denial aims at arguing against the carbon tax, which is grounded in a (political) belief that humans have a prominent role in the CONSTRUCTION of the *Greenhouse* (and are, therefore, expected to stop this CONSTRUCTION).

Alternatively, the dangerous levels of heat increases are exploited by online users who can refer to climate change as a series of threats caused by humans’ consumption. This unreasonable consumption is perceived as MISCHIEVOUS ACTIONS which must be reprimanded by a higher authority, as in the following:

(6) The global climate is changing. And it is rapidly morphing as the temperature rises too quickly. Over centuries, our dependence on carbon fuel is finally playing the death knell loud and clear. The overall greenhouse effect is causing a spike in the temperature of the Earth’s atmosphere and this is causing the polar caps to melt. Scientists anticipate that if the polar caps melted entirely, all the clear freshwater will mingle with the salty sea water to create a concoction that we couldn’t survive on at all. Rather, it will risk tainting the fresh water sources we still count on today. (EnTenTen, token: 6016353006)

In extract (6), the metaphor user pictures humans’ gas emissions as MISCHIEVOUS ACTIONS, which have long been unnoticed (‘finally’). However, the link established by scientists between emissions and climate change leads the metaphor user to infer that environmental threats represent a PUNISHMENT. The paradox highlighted in the extract between humans’ acknowledgement of such a link and their continuing reliance on gases is described as a LETHAL DEPENDENCE. The metaphor user does not consider the possibility of reduction and, therefore, does not question the impact of the ‘death knell’. I can also identify a conceptual association between the *greenhouse effect* and FOOD-related lexicon through the characterisation of the climatic effects as a DEADLY CONCOCTION. Here, the *Greenhouse* becomes a HEATED CONTAINER which imprisons humans following their misbehaviour. Such an imprisonment is followed by a series of lethal events – described in the extract as deliberate reprimands performed by a personified version of climate change – which constitute climate’s response to humans’ actions.

These three examples show that online discussions offer relevant data regarding the varying argumentative use of the metaphorical expression *greenhouse effect*. We see how scepticism may only be attributed to particular characteristics of the *greenhouse effect*, such as reduction policies. In the following section, I will discuss the argumentative uses of the metaphor in highly sceptical contexts.

## 6. Sceptical arguments

Sceptical newspaper articles are also of particular interest. This genre has been selected among other possible sceptical discourses because articles’ headlines allow the researcher to rapidly establish the sceptical stance. Here, I focus on how scepticism may alter humans’ place in the *Greenhouse*. I see that sceptical journalists favour a depiction of humans as the passive inhabitants of the *Greenhouse*: these views may either deny the existence of a dangerous phenomenon or question the possibility for humans to mitigate the evolution of the *greenhouse effect*. For instance, the example below emphasises the role of the planet in keeping humans’ habitat adapted to human life:

(7) The two scientists compared the predictions about what the atmosphere ‘should’ do to what satellite data showed the atmosphere actually did do during the 18 months before and after warming events since 2000. They found – shock! – that the computer models vastly *overestimated the greenhouse effect*. Turns out that *the Earth is far more capable of equalizing* its own temperature than environmentalists would have us believe. (*The New York Post*, Matt Paterson, 2 August 2011)

Extract (7) questions scientific findings related to the evolution of the *greenhouse effect*. The journalist uses scientific admitted overestimation to promote sceptical arguments related to humans’ contribution to dangerous warming. Indeed, the *greenhouse effect* is exclusively perceived in relation to climate change and, more precisely, to irreversible future climate change. As noted in our discussion of scientific examples, we see that these predictions are surrounded by uncertainties. In turn, the journalist depicts the *greenhouse effect* as a process, which does not bear any dangerous characteristics: according to the journalist, this effect will evolve without representing a threat to humans’ survival. I also see that possible human actions to mitigate the effect are not even mentioned. These actions are only inferred through a vague reference to ‘environmentalists’ who promote worrying views on the climate. Here, the Earth is the main protagonist of the narrative: through personification (‘is far more capable’), the planet is perceived as a benevolent character who is the sole entity capable of modifying the climate. Yet, as climate change may lead to its destruction, the journalist claims that the planet has a defensive system preventing catastrophic warming. The anthropogenic characteristic of the *greenhouse effect* is not explicitly denied: since the Earth is expected to ‘equalise’ its temperatures, I can infer that the journalist still considers humans’ alteration. However, the scepticism is related to the significance of humans’ alteration, which does not outreach the Earth’s capacities to provide adapted temperatures.

Scepticism may also be related to the reality of climate change as a phenomenon comprising temperature increase and anthropogenic causes. This is exemplified in the extract below:

(8) Flying into Churchill, the weather seems cold enough. If minus 5C means *the greenhouse effect is upon us*, heaven knows what it was like before. (*Daily Mail*, David Jones, 8 December 2007)

In extract (8), the journalist presents the *greenhouse effect* as a perceptible clue of climate change. Since the journalist cannot perceive such a clue (‘minus 5C’), he questions the very existence of the *Greenhouse* (‘upon us’). Here, the natural effect is not considered: the journalist only refers to the claims that humans have BUILT a *Greenhouse* which endangers their survival. Since this BUILT CONTAINER is characterised by warm temperatures, the experience of cold weather represents an event that leads sceptics to question the reality of this human action. In addition, the inference regarding the experience of temperate weather in the past (‘before’) is conceived as a proof that humans are not BUILDING A GREENHOUSE. Therefore, the sceptical stance is related to the existence of the *anthropogenic greenhouse*; in other words, the journalist disputes the fact that humans (or nature) have an active role in the modification of climate and that climate is changing.

We see that, in these two extract, the journalists mainly consider humans’ alteration and temperature increase. However, they do not focus on the lethal consequences of the phenomenon. These lethal consequences are described in other articles through the conceptualisation of the EARTH AS A HEATED CONTAINER. This is demonstrated in the example below:

(9) Many people are worried about the *greenhouse effect* of carbon dioxide. The real problem is that scientists are not telling the truth about carbon dioxide. Early in the earth’s history the earth was *boiling hot* and the major gas in the atmosphere was carbon dioxide. So the *greenhouse effect* of carbon dioxide isn’t as effective as supposed because with the extremely high amount of carbon dioxide in the atmosphere the earth cooled off. (*News release Wire*, 6 May 2005)

In extract (9), the journalist describes the *greenhouse effect* through references to an anthropogenic, climate change–related phenomenon (‘many people are worried about the greenhouse effect of carbon dioxide’) and references to a natural, non-threatening phenomenon (‘early in the earth’s history’). The existence of an unnatural *Greenhouse* BUILT by humans is denied: the ‘scientists’ quoted in the extract are identified as spreaders of fake news regarding the danger of humans’ alteration. First, I see that humans’ alteration is exclusively perceived through carbon pollution (‘greenhouse effect of carbon dioxide’). Yet, the journalist claims that humans do not play any role in the CONSTRUCTION of the *Greenhouse* since the MATERIAL they are adding (according to the scientists) was already present ‘early in the earth’s history’ (which might refer to a time when humanity did not exist). Second, the impact of this ADDITION OF MATERIAL (i.e. carbon dioxide) is not explicitly described as dangerous. I here see a problem in the journalist’s argumentation. Indeed, he or she first claims that the *greenhouse effect of carbon dioxide* was associated with threatening temperature increase (‘the earth was boiling hot’), but he or she denies that the *greenhouse effect* was the cause since it did not prevent the Earth from ‘cooling off’. Hence, even though the journalist aims at denying the impact of the *greenhouse effect*, he or she still endorses the fact that temperatures may significantly vary to the point that humans may not survive. In addition, from a cognitive viewpoint, another argumentative problem appears: the journalist denies the fact that the *greenhouse effect* has an impact on the Earth’s temperatures while the source domain GREENHOUSE still bears characteristics associated with a WARM CONTAINER. These argumentative issues appear because the journalist focuses on humans’ addition of carbon, and he or she argues that humans are only passive INHABITANTS who might suffer from uncontrollable climatic cycles. Yet, this occurrence still presents a threatening view on the climate which can ‘boil’ the Earth (contradicting extract 7). As a result, this lack of human control not only promotes human inaction towards carbon reduction (see also extract 5) but also emphasises the extent of the danger related to climate change.

These three extracts show that sceptical arguments in the media may focus either on the lack of real danger associated with the *greenhouse effect* – humans may not need to reduce their emissions – or on humans’ contribution to the BUILDING of the *Greenhouse* – natural climatic evolution represents a clue that humans cannot control the climate. In the following section, I will further discuss the different roles attributed to humanity in the three genres.

## 7. Discussion

Considering existing research on the *greenhouse effect* metaphor, this article demonstrated the variety of characteristics associated with source domain GREENHOUSE in climate change discourse. Depending on the stance adopted in the texts, I see that different genres attribute different roles to humans in the *Greenhouse*. Therefore, metaphors which are constitutive of climate change theories cannot be attributed a single, scientific meaning (Deignan et al., 2019): this meaning is exploited by different discourse producers to promote environmental arguments (Musolff, 2016).

Considering Romaine’s (1996) finding about the identification of HUMANS AS PLANTS CONTAINED IN THE GREENHOUSE, I see that scientific uses and online discussions do not significantly endorse such a characterisation, even when they promote arguments questioning climatic claims (in online forums). However, this mapping is relevant to sceptical journalists who criticise claims related to humans’ alteration of the *Greenhouse*. The GREENHOUSE concept allows PLANTS to grow but PLANTS are not expected to have any influence on the GREENHOUSE that contains them. In addition, PLANTS are non-human, inanimate, and material referents. These HUMANS-PLANTS therefore correspond to humans’ passive role in the *Greenhouse* narrative. Sceptical journalists relied on the mapping HUMANS AS PLANTS to convince recipients not to adopt environmental policies and not to feel threatened by the phenomenon. Alternatively, in online forums, users may argue against certain policies (i.e. the carbon tax) but still endorse the image of humans contributing (yet, partially) to the CONSTRUCTION of the *Greenhouse*.

While the GREENHOUSE concept was figuratively extended to become an EARTH-CONTAINER providing adapted temperatures to its CONTENT, the fact that pollution has altered the climate means that the GREENHOUSE does not provide adapted temperatures anymore. This subsequently alters the identification of HUMANS AS PLANTS. Humans are conceptualised as the INADEQUATE CONTENT OF THE GREENHOUSE, following appropriate knowledge about the characteristics of PLANTS, which grow within a *greenhouse*, and the characteristics of PLANTS, which do not grow in a *greenhouse*. The impact of humans’ activities has promoted a different view on humans’ role in the *Greenhouse*. This active role may slightly differ in scientific papers and in online discussions.

First, in scientific extracts, pollution is perceived as a MATERIAL added by humans to the *Greenhouse*. This MATERIAL implicitly demonstrates humans’ contribution to the BUILDING of the GREENHOUSE. In online discussions, humans are also identified as the BUILDERS, but this process may or may not have any incidence on the *Greenhouse* – which can still be described through its natural characteristics.

Second, in scientific extracts, humans are attributed a prominent role: they are not only represented as the BUILDERS of the *Greenhouse* – leading to scientific arguments regarding the alteration of living conditions – they are also depicted as the DESTROYERS of the natural *Greenhouse* which provided adapted temperatures. This conceptual DESTRUCTION in scientific descriptions of the *greenhouse effect* is related to an emphasis on the threatening consequences of human activities, which may lead to irreversible climate change. Hence, the claim that scientists may not promote threatening images of climate change (Weingart et al., 2000) can be contradicted by my findings: I showed that scientists’ documented view on the phenomenon enables them to offer a detailed metaphorical picture of humans’ alteration of the environment. In addition, my findings echo existing literature about scientific representations of humanity: I see that scientists depict humans as a species capable of altering the climate. Yet, while they draw an explicit distinction between a natural, anthropogenic or stratospheric *Greenhouse*(*s*), such a distinction does not occur regarding humanity. Humanity – as a whole – contributes to the (DE)CONSTRUCTION, discarding individual or national pollution rates (see Besley et al., 2018; Dawson, 2018). Scientists dispute the image of humans as PLANTS as they aim to demonstrate humans’ active role not only in the alteration of climate but also in the application of solutions.

The threat represented by future climate change – which transforms into ‘climate chaos’ (Dahl and Fløttum, 2014: 415) – has promoted particular metaphorical descriptions. These descriptions favour metaphorical pictures of a former NATURAL GREENHOUSE-EARTH which has fully achieved its transformation into a HEATED CONTAINER. Such a picture of hypothesised evolutionary trends may contradict the scientific stance which focuses on experiment, models, and tests (Knudsen, 2015). Online users and sceptical journalists relied on this mapping to identify a danger which may either be a direct response from the climate to humans’ activities or represent the uncontrollable characteristic of the climate, discarding humans’ presence in the *Greenhouse*. The role of humans as the powerless CONTENT promotes a re-occurrence of the identification of HUMANS AS PLANTS. Yet, this view on humans’ passive role is not endorsed in scientific descriptions. I can see how the metaphor may be used to fit scientific observations, and how the metaphor has been extended in argumentative discourses to produce metaphorical conceptualisations resulting in non-scientific descriptions of climate change. This discussion highlights a graduation from the prominent roles attributed to humans advertised in science, the questioning and endorsement of certain characteristics related to these roles in forums, to the promotion of the negligible role played by humans in sceptical articles.

I should finally acknowledge that my corpus-based approach (Tognini-Bonelli, 2001) does not allow me to make any exhaustive claims about scientific, journalistic or online uses of climate change metaphors. Yet, the examples discussed in this article demonstrate the varying places attributed to humans through the uses of a specific metaphor, depending on particular stances on climate change. In addition, the selection of particular sources (*Nature, EnTenTen, Nexis*) does not allow me to offer a diachronic view on metaphorical descriptions. It would be relevant to use comparable sources for the corpus to observe how the metaphor has evolved over time. For instance, forthcoming research will focus on the impact of controversial events on the use of metaphors in different discourses about climate change. This article demonstrated that questioning climate change can lead to the questioning of humans’ environmental capacities. Through the use of a metaphor, environmental control may be attributed to human agents or to a personified version of the ecosystem.

## Notes

1. The Climategate scandal took place in 2009 when climate scientists from the University of East Anglia saw their emails hacked and spread to the media. The content of these emails highlighted scientists’ doubts and pressures, and this impacted the public opinion: the discussion of this scandal in the media promoted a rise of scepticism.

2. Extract (3) questions the mapping of the source domain RUNAWAY to the target domain EARTH CLIMATE. Here, the scientist argues that the target domain VENUS CLIMATE is more appropriate.

3. Edward Parson’s biography is available at: https://www.ucsusa.org/about/people/edward-parson
